# Accessing Physical Activity and Health Disparities among Underserved Hispanic Children: The Role of Actual and Perceived Motor Competence

**DOI:** 10.3390/ijerph17093013

**Published:** 2020-04-26

**Authors:** Tao Zhang, Joonyoung Lee, Tsz Lun (Alan) Chu, Changzhou Chen, Xiangli Gu

**Affiliations:** 1 Department of Kinesiology, Health Promotion and Recreation, University of North Texas, Denton, TX 76203, USA; 2 Department of Psychology, University of Wisconsin-Green Bay, Green Bay, WI 54311, USA; 3 School of Leisure Sport, Shanghai University of Sport, Shanghai 200438, China; 4 Department of Kinesiology, University of Texas at Arlington, Arlington, TX 76019, USA

**Keywords:** motor competence, perceived competence, physical activity, quality of life, Hispanic children, low-income families, clinical intervention strategies

## Abstract

Promoting physical activity (PA) and eliminating health disparities among underserved minority children is a public health priority. The main purpose of this study was to examine the relationship of actual motor competence (a set of object control skills) and perceived motor competence with PA participation and health-related quality of life (HRQoL) among underserved Hispanic children who were born in the U.S. Guided by Stodden et al.’s conceptual model, we tested the direct and indirect effects (mediational model) of actual motor competence on health-related outcomes (PA and HRQoL) through perceived motor competence. Participants were 215 underserved Hispanic children (*M*_age_ = 10.55 years, *SD* = 0.53 [age range 10–12]; 51.6% boys), recruited from four elementary schools in the southwestern U.S., who completed validated questionnaires assessing their perceived motor competence, PA, and HRQoL. Their actual motor skills were assessed using PE Metrics^TM^. After examining the associations among the variables, we tested the hypothesized model using structural equation modeling (SEM; AMOS 25). The hypothesized model indicated a good fit (χ²/df = 38.427/24 = 1.60 < 5; non-normed fit index (NFI) = 0.93; comparative fit index (CFI) = 0.968; root mean square error of approximation (RMSEA) = 0.053 [0.016, 0.083]). The effect of actual motor competence on PA and HRQoL was fully mediated by perceived motor competence. The findings demonstrated the mediating role of perceived motor competence between actual motor competence and health-related outcomes (PA and HRQoL) among underserved Hispanic children. The results highlight that actual motor competence significantly predicted underserved Hispanic children’ perceived motor competence, which in turn positively predicted their PA and HRQoL. These findings have significant practical implications for future intervention strategies of randomized clinical trials in schools aimed at promoting PA and HRQoL and eliminating health disparities among underserved Hispanic children.

## 1. Introduction

Promoting physical activity (PA) is an essential component in health-promotion policies with regard to the prevention of childhood obesity and the promotion of children’s overall well-being [1,2]. Regular PA participation can lead to numerous health benefits, such as enhancing cardiorespiratory and cognitive functioning and decreasing depression risks [1,3]. As a subset of quality of life, health-related quality of life (HRQoL) measures individuals’ perceptions of their health. HRQoL includes physical and psychosocial functioning [4], which is one of the essential components of health-related outcomes of diverse clinical interventions for practitioners, researchers, and patients [5]. Previous research has demonstrated that childhood obesity, as a result of physical inactivity, is associated with lower HRQoL [6,7] and depression [8], and that greater PA participation is associated with higher HRQoL among school-aged children [5,9,10]. 

As the fastest-growing ethnic minorities in the U.S., Hispanic children from low-income families have been identified as a high-risk group for being obese, showing less PA and high sedentary behaviors [11]. Specifically, 46.2% of Hispanic children, aged 6–11 years, are categorized as overweight or obese, compared to 29.4% of White children, 38.1% of Black children, and 19.9% of Asian children [12]. A longitudinal study showed a consistent decline in moderate-to-vigorous PA (MVPA) and an increase in sedentary behaviors among Hispanic children aged 8–11 years [13]. Furthermore, although the health benefits of regular PA participation have been well documented, over half of the young children do not meet recommended PA guidelines (i.e., 60 minutes of daily MVPA) in the U.S.; the rate is higher among Hispanic children [14,15]. Promoting PA and eliminating health disparities among underserved Hispanic children from low-income families has become a public health priority [16,17].

Motor competence (i.e., actual competence in motor skills) is a behavioral determinate of healthy weight and lifetime PA participation during childhood [18,19,20]. Stodden and colleagues [18] proposed a conceptual model suggesting a reciprocal relationship between actual motor competence and PA, with perceived motor competence mediating this relationship from early to late childhood. This conceptual model indicates that children with less developed/skilled motor competence may show disengagement in PA, leading to a greater risk of being obese across age [18]. Empirical evidence has demonstrated that actual motor competence influences children’s physical and psychosocial development [21,22,23,24]. A longitudinal study demonstrated that a child who is more skilled in object control skills, but not locomotor skills, is more likely to become a physically active adolescent compared to their peers [25]. Further, Barnett and colleagues [25] suggest that children’s motor competence, particularly in object control skills (e.g., striking, dribbling, throwing), is important for PA participation later during adolescence. Mastery of object control skills plays a vital role in increasing opportunities to participate in PA, such as sports, games, and free play [26]. For example, it is important to practice and master dribbling skills for a child to engage in basketball with peers in a game-like situation. To be confident in playing Tee-ball on a team, children need to gain basic knowledge and perceptions of striking skills. 

Researchers suggested that both actual and perceived motor competence are critical predictors of long-term PA and health-related outcomes in children and adolescents [27,28]. Recent evidence noted that perceived motor competence served as a mediator between actual motor competence and PA among children in 8- to 9-year-old Iranian girls [29]. Gu and colleagues [30] provided preliminary evidence regarding the indirect effect of actual motor competence on PA participation through perceived motor competence among U.S. children. Childhood is an important period to establish the foundation for motor competence, including locomotor (e.g., running, sliding, jumping) and object control skills (e.g., striking, dribbling, throwing) [19,20]. It has been recommended that children should to build sufficient knowledge, skills, and confidence to engage in developmentally appropriate activities, especially in the transition period from later childhood to adolescence (e.g., 4th and 5th grade) [30,31].

**Figure 1:**
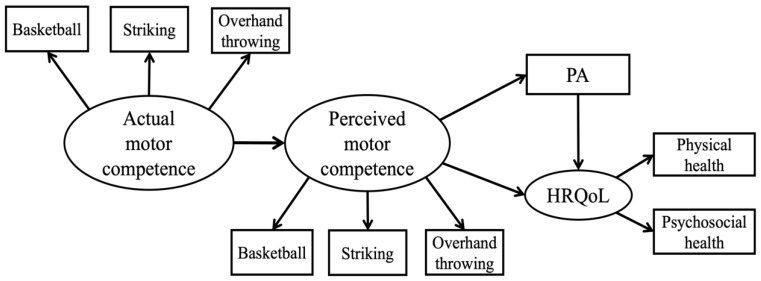
Hypothesized model of this study. PA = physical activity, HRQoL = health-related quality of life.

Hispanic children from low-income families have a high risk of delay in developing fundamental motor skills (FMS), especially object control skill competence [32,33]. Although school physical education (PE) provides good opportunity for children to practice skills, the perceived barriers and limited community resources (e.g., safety issue, lack of accessibility to culturally appropriate facilities and programming, cost) were observed in underserved Hispanic communities [1,34,35], which may indirectly result in health disparities and delayed FMS development in childhood. In this study, we mainly assessed the actual motor competence by solely focusing on object control skills (i.e., basketball, striking, and overhand throwing) and how those skills correlated with perceived motor competence and health-related outcomes (i.e., PA and HRQoL) among a sample of Hispanic children from low-income families. Hence, the main purpose of this study was to examine the relationship of actual motor competence (a set of object control skills) and perceived motor competence with PA participation and HRQoL among underserved Hispanic children. We hypothesized that the relations of actual motor competence and health-related outcomes (i.e., PA and HRQoL) would be completely or partially mediated by perceived motor competence. Guided by Stodden et al.’s conceptual model, we tested the direct and indirect effects (mediational model) of actual motor competence on health-related outcomes through perceived motor competence (see Figure 1). 

## 2. Methods

### 2.1. Participants

Participants were 215 Hispanic students (*M*_age_ = 10.55 years, *SD* = 0.53 [age range 10–12]; 51.6% boys) from four elementary schools in the southwestern United States. The four elementary schools were located in the same suburban school district with more than 45% minority and 78% from low-income families (i.e., receiving free or reduced lunch). Prior to the start of the study, the University of North Texas’s Institutional Review Board approved the study protocol (project identification code: 13224-R15), and the permission to collect data was received from the school district and each school’s principal. All fourth- and fifth-grade Hispanic children who were born in the U.S. from low-income families with free or reduced lunch status identified by four elementary schools were invited to participate in this study. Children with disabilities that prohibit PA were excluded from the study. We did not identify any medical conditions associated with motor clumsiness such as Asperger’s syndrome or Down syndrome in the present study.

### 2.2. Procedure

Participants were recruited with the assistance of each school’s principal, and the school provided information about children’s race/ethnicity, so low-income Hispanic children could be identified and invited to participate in this study. Parental informed consents and student assents were collected by classroom teachers. After receiving both forms, our research team gathered data during three PE classes within a three-week period. The research assistants, who completed two training sessions on the research protocols, helped participants complete validated paper-and-pencil surveys assessing perceived motor competence, PA, and HRQoL. Our research team specifically instructed and explained each questionnaire item to the participants to ensure their understanding by reading it out loud. If participants had less proficiency in reading English, we provided a Spanish version of the questionnaires. All participants were informed that their responses to the answers would remain confidential and anonymous to their teachers to encourage them to answer truthfully. 

### 2.3. Measures

#### 2.3.1. Actual Motor Competence

The PE Metrics^TM^ was used to measure children’s actual motor competence, with three object control skill assessments (elementary fifth-grade level; National Association for Sport and Physical Education, 2010; [36]). In line with the purpose of this study and the PE teachers’ recommendations, two research assistants assessed the Hispanic children’s actual object control skills (i.e., basketball, striking, and overhand throwing). Before the assessments, the research assistants provided the children with specific instructions for each object control skill and demonstrated the skill. Each PE Metrics^TM^ motor skill rubric has its own criteria (the forms of movement and continuous action), and each skill was scored based on a four competence-level scoring rubric (4 = *consistently*; 3 = *usually*; 2 = *sometimes*; 1 = *seldom*). Considering that the discrepancies between the two research assistants’ ratings were no more than one, the average ratings between the two were used; if the difference was more than one, the children were made to complete one more trial. Table 1 indicates each of the three object control skills in the PE Metrics^TM^ [36]. The intraclass correlation coefficients (ICC) indicated that actual object control skill measures were reliable with satisfactory ICC (= 0.75; [37]). The PE Metrics^TM^ is a reliable and valid national standard-based assessment tool to assess elementary (K–5th grade) students’ actual motor competence [35,38]. 

#### 2.3.2. Perceived Motor Competence

To assess the children’s perceived motor competence, a 12-item Perceived Competence Scale (PCS) was used [39]. The original PCS is a short, four-item questionnaire, and is one of the most face valid instruments designed to assess constructs based on self-determination theory [40]. We modified this scale (4 items × 3 = 12 items) for overhand throwing, striking with a paddle, and basketball skills (dribbling, passing, and receiving). For example, the sample questions about overhand throwing include, “I feel confident in my ability to do overhand throwing”, “I feel capable of doing overhand throwing now”, “I am able to do overhand throwing now”, and “I feel able to meet the challenge of doing overhand throwing”. The items were rated on a seven-point Likert-type scale (i.e., 1 = *not at all* and 7 = *very true*). The means of every four items were taken to give an indication of the magnitude of children’s perceived motor competence in overhand throwing, striking with a paddle, and basketball skills (dribbling, passing, and receiving), respectively. Cronbach’s alpha reliability coefficients for overhand throwing, striking with a paddle, and basketball skills (dribbling, passing, and receiving) were 0.83, 0.85, 0.90, indicating acceptable internal consistency in the present study.

#### 2.3.3. Physical Activity

The self-reported Physical Activity Questionnaire for Older Children (PAQ-C, Kowalski et al. 2004; [41]) was used to measure participation in different activities during PE, lunch break, and recess time, over the past seven days [41]. The PAQ-C includes nine items (i.e., each scored on a five-point scale; 1 = *low* and 5 = *high*), and the mean scores of all items are calculated, which indicates the PAQ-C activity summary score. PAQ-C has been shown to have high validity and reliability of general PA levels among school-aged children (ICC = 0.96) [42], and this scale had an adequate internal reliability coefficient in the present study (Cronbach’s α coefficient = 0.73). 

#### 2.3.4. Health-Related Quality of Life (HRQoL)

Children’s HRQoL was measured using the self-reported Pediatric Quality of Life Inventory^TM^ (PedQL^TM^ 4.0, Varni et al., 2001; [4]), which includes 23-items: physical health (8 items) and psychosocial health (15 items). Children were asked to indicate how much of a problem each item had been during the past one month, on a five-point scale ranging from “*never*” (0) to “*always*” (4). The sample items were “It is hard for me to do the sport activity or exercise” in physical health and “It is hard to pay attention in class” in psychosocial health. To designate higher scores with a higher HRQoL, responses to HRQoL items were reverse scored and linearly transformed to a 0–100 scale (0 = 100, 1 = 75, 2 = 50, 3 = 25, and 4 = 0). The scores for physical health and psychosocial health were averaged correspondingly. The adequate validity and reliability of the questionnaires were identified by Varni and colleagues [4]. Cronbach’s alpha reliability coefficients demonstrated acceptable reliability for underserved children in this study (Cronbach’s α; physical health = 0.80 and psychosocial health = 0.83). 

### 2.4. Data Analysis

SPSS 25.0 (IBM Corp., Armonk, NY, USA) was used to calculate the descriptive statistics of all variables. Correlation coefficients were then computed to explain the bivariate associations among actual motor competence (i.e., basketball, striking, and overhand throwing), perceived motor competence (i.e., basketball, striking, and overhand throwing), and PA and HRQoL (i.e., physical and psychosocial health) among underserved Hispanic children. Further, we tested the hypothesized model using structural equation modeling (SEM; AMOS 25, [43]). The model was determined as a good fit using the following criteria [44,45]: the chi-square test, root mean square error of approximation (RMSEA < 0.08), non-normed fit index (NFI ˃ 0.90), and comparative fit index (CFI ˃ 0.90). Lastly, the bootstrapping procedure was conducted to assess the statistical significance of the indirect effects (mediation model) by calculating the 95% confidence intervals (95% CIs; [46]). An indirect effect is considered significant when the 95% CI does not include zero. 

## 3. Results

As described in Table 2, all study variables were normally distributed (skewness and kurtosis between −2 and +2; [47]), except for physical health of HRQoL. Further, the mean scores for all variables were generally above the moderate range, indicating that the students’ actual motor competence (i.e., basketball, striking, and overhand throwing) and perceived motor competence (i.e., basketball, striking, and overhand throwing), PA, and HRQoL were all relatively high.

The correlations among the study variables are presented in Table 3. Basketball, striking, and overhand throwing, which reflected actual motor competence, were positively associated with all three components of their perceived motor competence for basketball, striking, and overhand throwing, respectively (rs ranging from 0.21 to 0.61, *p* < 0.01), but not associated with PA, physical health, and psychosocial health. Interestingly, a weak but significant correlation between physical health and overhand throwing was observed (r = 0.16, *p* < 0.05). Three components of perceived motor competence were all significantly associated with PA (rs ranging from 0.34 to 0.61, *p* < 0.01), and PA was significantly associated with physical health and psychosocial health components of HRQoL (rs ranging from 0.20 to 0.56, *p* < 0.01), respectively.

**Figure 2:**
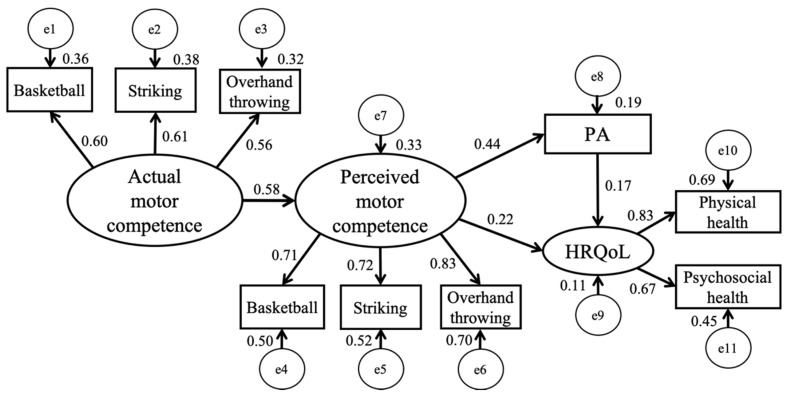
Final structural equation model of this study. PA = physical activity, HRQoL = health-related quality of life.

Before testing the hypothesized structural model, the measurement model was first constructed to estimate the latent variables, and results showed a good fit to the data (χ2/df = 27.617/17 = 1.62, *p* < 0.05; NFI = 0.936; CFI = 0.974; RMSEA = 0.05; 90% CI [0.002, 0.089]). Then, the follow-up SEM was conducted to test the direct and indirect effects of actual motor competence on PA through perceived motor competence. The structural model also produced a good fit to the data according to the various indices of fit: χ²/df = 38.427/24 = 1.60; NFI = 0.93; CFI = 0.968; RMSEA = 0.053 [0.016, 0.083] (See Figure 2; [48,49]). Specifically, the model accounted for 33.0%, 19.0%, and 11.0% of the variance in perceived motor competence, PA, and HRQoL, respectively. Path coefficients suggested that actual motor competence (β = 0.58, *p* < 0.01) was directly and positively associated with perceived motor competence, which significantly predicted PA (β = 0.44, *p* < 0.01) and HRQoL (β = 0.22, *p* < 0.05). The direct effect from actual motor competence to PA and HRQoL was not significant in this study. Thus, it suggests that the effect of actual motor competence on PA (95% CI = [0.158, 0.376]) and HRQoL (95% CI = [0.041, 0.312]) was fully mediated by perceived motor competence in this population.

## 4. Discussion

Promoting PA and eliminating health disparities among underserved Hispanic children is a public health priority. The present study aimed to examine the relationship of actual motor competence (a set of object control skills) and perceived motor competence with PA and HRQoL among underserved Hispanic children from low-income families. Consistent with the hypothesized model, the results provided support for Stodden et al.’s conceptual model. More importantly, this study extended the predictive utility of the model by assessing health-related outcomes (i.e., HRQoL) among underserved minority children. As a comprehensive and multidimensional construct of pediatric mental health, HRQoL is frequently being used to assess children’s physical health and psychosocial health. In congruence with previous studies, the results of this study confirmed a positive relationship between PA and both components of HRQoL, which suggests an important role of school-based PA programs (i.e., recess, PE) on health-related outcomes among underserved Hispanic children. Given the fact that underserved children have lack of community resources, recent studies suggest that creating a school-based physical activity environment can highly benefit underserved Hispanic children [16,17]. Additionally, when the school offered quality physical and social PA environments, underserved Hispanic children tended to put more effort into PE and engage in PA that can contribute to improving their actual motor skill competence [16]. Understanding the roles of actual and perceived motor competence in predicting underserved Hispanic children’s health-related outcomes such as PA and HRQoL can provide meaningfully practical implications for future randomized clinical interventions in schools aimed at promoting PA and eliminating health disparities [50]. 

The results suggest a positive association between actual motor competence and perceived motor competence among underserved Hispanic children from low-income families. This finding for actual motor competence is consistent with the existing positive link with perceived motor competence in different race groups, including in White [51], Black [52,53], and Asian children [54]. The findings further revealed that actual competence in object control skills was not directly associated with PA and HRQoL, which is in contrast to previous studies showing direct and positive effects of actual motor competence on PA [27,51] and HRQoL [21]. Such contradictory results could be partially explained by the fact that previous studies used a different method to measure PA (using the objective PA measurement; [51], and examined a different age group (*M*_age_ = 16; [27], *M*_age_ = 5.37 [21]). More empirical studies are needed to provide a more comprehensive understanding of the roles of actual motor competence and health-related outcomes among underserved children. 

It is promising that the results support that perceived motor competence can be enhanced at this age group when they are skillful and able to accomplish different movement tasks especially for object control skills, which in turn may contribute to promoting children’s PA and HRQoL [51,55]. The SEM results support the indirect effect of actual motor competence on PA through perceived motor competence, which is in line with a previous study in the adolescent population [27]. Similarly, a study involving Danish children found children with higher actual motor competence and perceived motor competence demonstrated a higher level of motivation to engage in sports and had better mental health outcomes compared to their peers [55]. Findings from a longitudinal study also noted that perceived motor competence during childhood strongly predicted adolescent PA and fitness [56]. Nevertheless, improving both actual and perceived motor competence should be considered in childhood, because the combined effects may contribute to health promotion from a public health perspective and may reduce the trajectory of childhood obesity among this underserved minority population [51,57]. Improving both actual and perceived motor competence suggests that it is important to develop effective health promotion programs in school setting (i.e., PE, recess) focusing on mastering object control skills within a supportive learning environment (i.e., build perceived competence) among underserved Hispanic children in order to promote their health-related outcomes. 

This study not only provided us with meaningful evidence to understand the roles of actual and perceived motor competence on PA and HRQoL among underserved Hispanic children, but also provided insightful recommendations of intervention strategies for school PA specialists and health promoters such as PE teachers and health coordinators to enhance underserved Hispanic children’ health-related outcomes. Specifically, with necessary training and professional development opportunities, school PA specialists and health promoters may design developmentally appropriate PE and recess programs to promote students’ actual motor skills in schools [23,58], especially age-appropriate object control skills such as basketball, striking, and overhand throwing. In doing so, students may gain additional opportunities to learn and practice those ball skills and be more likely to involve in sports and games in their leisure time. It is also recommended to design culturally relevant activities (e.g., basketball, baseball, football) that could motivate and engage underserved Hispanic children to learn and practice those skills, which may enhance their perceived competence of performing various sport skills. Such efforts can address the health and PA disparity issues in underserved Hispanic children [16]. Last but not least, school PA specialists and health promoters may provide a learning environment emphasizing self-improvement instead of peer comparison for children’s goal-setting and skill practices in PE and recess programs [10]. 

To our knowledge, this was the first study to examine the combined effects of both actual and perceived motor competence on minority Hispanic children’s PA by using an aligned instrument solely focused on object control skills [59]. Another unique contribution of this study was employing SEM to test a hypothesized model of actual and perceived motor competence on underserved Hispanic children’s PA and HRQoL. However, this study has several inherent limitations that need to be addressed for future research. First, this study used a cross-sectional research design in which causality could not be established. Future research should consider using an experimental research design or a longitudinal research design to examine the associations among the study variables in this minority population. Such efforts would provide a better understanding of causality for those hypothesized models. Second, children’s PA was measured using a subjective measure. Although children’ leisure time PA was fully captured by the self-reported scale, future studies may also assess children’s PA using accelerometers or pedometers. Third, given the fact that sex can be an important moderator among actual motor competence, perceived motor competence, PA participation, and HRQoL among underserved Hispanic children, examining sex differences warrants further investigation in future endeavors. Finally, the results might not be generalized beyond other ethnic minority children in different regions (e.g., rural and urban). Future studies should further consider investigating underserved minority children in different regional settings because children’s motor skills, PA, and health-related outcomes can be affected by physical environmental variables from the ecological perspective [60,61]. 

## 5. Conclusions

The findings of this study demonstrate the mediating role of perceived motor competence between actual motor competence and health-related outcomes (PA and HRQoL) among underserved Hispanic children from low-income families. The results highlight that actual object control skills competence significantly predicted underserved Hispanic children’s perceived motor competence, which in turn positively predicted their PA and HRQoL. These findings have significant practical implications for future intervention strategies of randomized clinical trials in schools aimed at promoting PA and eliminating health disparities among underserved Hispanic children. Based on these findings, PA specialists and health promoters should provide sufficient opportunities for underserved Hispanic children to engage in activities for practicing age-appropriate object control skills such as basketball, striking, and overhand throwing. The findings support the theoretical tenets of the Stodden and colleagues’ conceptual model and extend empirical evidence to support the potential role of the combined effects of actual and perceived motor competence on PA and HRQoL among underserved minority children. 

## Figures and Tables

**Table 1:** A score range for each criterion and total score of three object control skills in physical education (PE).

Performance	Criterion	Total Score
Basketball dribbling, passing, and receiving	Dribbling (0–4) Passing (0–4) Receiving (0–4)	0–12 (9 = competent)
Striking with a paddle (one trial)	Form (0–4) Continuous strikes (0–4)	0–8 (6 = competent)
Overhand throwing	Form (0–4; 1st, 2nd, 3rd) Accuracy to target (0–4; 1st, 2nd, 3rd)	0–24 (18 = competent)

**Table 2:** Descriptive analysis for the study variables (*N* = 215).

Variables	Range	*M*	*SD*	Skewness	Kurtosis
1. Basketball	3.5–12	9.35	1.92	−0.66	−0.02
2. Striking	2–8	5.31	1.86	−0.19	−1.1
3. Overhand Throwing	6–24	16.49	3.81	−0.16	−0.28
4. PMC-Basketball	1–7	5.31	1.63	−0.99	0.18
5. PMC-Striking	1–7	4.43	1.59	−0.25	−0.64
6. PMC-Overhand Throwing	1–7	5.22	1.44	−0.58	−0.44
7. Physical Activity	1.52–4.86	3.15	0.66	−0.15	−0.10
8. Physical Health	0–100	81.88	1.13	−1.5	3.47
9. Psychosocial Health	18.33–100	74.96	1.03	−0.95	0.99

Note. *M* = mean, *SD* = standard deviation, PMC = perceived motor competence.

**Table 3:** Correlations among the study variables (*N* = 215).

Variables	1	2	3	4	5	6	7	8	9
1. Basketball	—								
2. Striking	0.36 **	—							
3. Overhand Throwing	0.34 **	0.34 **	—						
4. PMC-Basketball	0.26 **	0.21 **	0.22 **	—					
5. PMC-Striking	0.28 **	0.39 **	0.26 **	0.46 **	—				
6. PMC-Overhand Throwing	0.28 **	0.28 **	0.27 **	0.61 **	0.60 **	—			
7. Physical Activity	0.07	0.07	0.06	0.36 **	0.37 **	0.34 **	—		
8. Physical Health	0.04	0.01	0.16 *	0.26 **	0.17 *	0.18 **	0.21 **	—	
9. Psychosocial Health	−0.04	0.03	0.03	0.11	0.15 *	0.15 *	0.20 **	0.56 **	—

Note. * *p* < 0.05; ** *p* < 0.01, PMC = perceived motor competence.
